# Relationship between serum interleukin-6 levels and severity of coronary artery disease undergoing percutaneous coronary intervention

**DOI:** 10.1186/s12872-023-03570-8

**Published:** 2023-11-28

**Authors:** Nadia Bouzidi, Habib Gamra

**Affiliations:** 1 Cardiothrombosis Research Laboratory, LR12SP16 University of Monastir, Monastir, 5000 Tunisia; 2 Laboratory of Human Genome and Multifactorial Diseases, Faculty of Pharmacy of Monastir, University of Monastir, LR12ES07 Monastir, Tunisia; 3 Cardiology A Department Fattouma, Bourguiba University Hospital, Monastir, 5000 Tunisia

**Keywords:** IL-6, Coronary artery disease, STEMI, Severity, Gensini score

## Abstract

**Background:**

Cytokines play a potential role in atherosclerosis pathogenesis and progression. We investigated the association of interleukin-6 (IL-6) with the angiographic severity of coronary artery disease (CAD).

**Methods:**

Three hundred ten angiografically diagnosed CAD patients and 210 controls were enrolled in this study. CAD patients were stratified according to IL-6 cut-off value into high levels IL-6 group (≥ 9.5 pg/mL) and low levels IL-6 group (< 9.5 pg/mL). The severity of CAD was assessed according to Gensini score (GS), artery stenosis degree and the number of vessels involved. The mean age was 60.3 ± 11.0 years.

**Results:**

The level of IL-6 in patients was increased compared to controls and ranged from 1.5 to 3640.0 pg/mL. High levels of IL-6 were significantly associated with high levels of GS (> 40) but not with stenosis degree and vessel score. GS levels were significantly more elevated in patients with high levels of IL-6 group than in low IL6 levels patients (60.6 ± 39.5 *vs* 46.7 ± 37.2; *p* = 0.027). The analysis of the ROC curve performed in myocardial infarction patients showed that IL-6 (AUC: 0.941 (CI 95% 0.886, 0.997; *p* < 0.001) could be a powerful predictor marker in evaluating the infarct size after myocardial infarction when compared to myonecrosis biomarkers.

**Conclusions:**

IL-6 levels were associated with the severity of CAD assessed by the GS. Based on the highest levels of IL-6 measured in patients with STEMI, our study strongly suggests that IL-6 could be a powerful marker in evaluating the myocardial necrosis.

**Trial registration:**

ClinicalTrials.gov Number: NCT03075566 (09/03/2017).

## Introduction

Atherosclerosis, an inflammatory process, is characterized by the involvement of multiple risk factors and the complexity of its pathophysiology [1]. The pathogenesis of the disease involves several stages: once stimulated, the damaged vascular endothelial cells secrete various pro-inflammatory molecules that mediate recruitment, migration, cell proliferation and regulation of protein and lipid synthesis. Macrophages internalize oxidized LDL and subsequently differentiate into foam cells [2]. Activation of macrophages causes acute inflammation and secretion of growth factors. Cytokines secreted by activated pro-inflammatory cells induce thickening or rupture of atherosclerotic plaques and arterial thrombosis [3]. The process of atherosclerosis follows well-defined steps and can affect all sites of coronary artery [4]. This pathophysiological mechanism induces the formation of an important lipid necrosis, a cascade of inflammatory reactions [5], alteration of the cascade of coagulation and fibrinolysis [6]. This mechanism causes the release of various cytokines, which are involved in the destruction of the fibrous cap and plaque instability leading to SCA [7]. IL-6, a pleiotropic cytokine intervenes in the acute phase of chronic inflammation response. IL-6, a member of the IL family, plays a proinflammatory role inducing the adhesion and aggregation of inflammatory cells throughout the organism which explains its involvement in the development of atherosclerosis and thrombosis [8]. Blood IL-6 levels are associated with CVD as including endothelial dysfunction, arterial stiffness and atherosclerosis [9]. With regards to CAD risk prediction, we conducted this prospective study to evaluate the association between serum IL-6 levels with the severity of CAD in patients with acute coronary syndrome, as confirmed by elective coronary angiography and Gensini score.

## Materials and methods

### Methods

#### Patients

We enrolled 207 blood donors subjects in the blood bank department in the Tahar Sfar University Hospital of Mahdia as controls in this study. Consecutive patients complaining of chest pain who underwent invasive coronary angiography for the diagnosis of CAD. A total of 310 CAD patients recruited from the Department of Cardiology, University Hospital (Monastir, Tunisia) showed by angiography at least one coronary stenosis of more than 50% of the luminal diameter that were included in the study. We followed the methods of Bouzidi N et al. 2020 [10].

The diagnosis of ACS was based on the guidelines of the European Society of Cardiology (ESC) [11]. NSTEMI-Acute coronary syndrome (ACS) included NSTEMI and UA. NSTEMI was defined as elevated cardiac troponin I (cTnI) values without new ST elevation on electrocardiogram with ischemic symptoms. UA was defined as having newly developed and accelerated chest symptoms on exertion or rest angina without the detection of signifcant cTnI increase. STEMI was defined as a persistant electrocardiographic ST-segment elevation associated to high levels of cTnI after chest pain symptoms (> 20 min).

Chronic lung disease, liver disease, kidney disease, concomitant inflammatory diseases such as infections and autoimmune disorders, or malignancy were excluded. The study protocol complied with the Declaration of Helsinki, and this study protocol was reviewed and approved by the National Committee for Medical and Research Ethics of Farhat Hached University Hospital (Sousse, Tunisia). Written informed consent was obtained from each subject.

#### Angiographic severity

Coronary angiographic data were collected from patient catheterization laboratory records and reviewed independently by interventional cardiologists. The severity of CAD was ascertained by the degree of epicardial coronary artery stenosis and was classified as previously published guidelines, as moderate (50–70% stenosis), and severe (> 70% stenosis) and multivessel disease extent [1, 2 or 3-vessel disease stenosis (> 50%)]. Accordingly, patients were categorized as having one or multi-vessel disease. Multi-vessel CAD was confirmed when at least two of the major coronary arteries had significant atherosclerosis [1].

Coronary lesion severity was assessed in each patient by the Gensini Score (GS) [12], which was calculated by scoring each atherosclerotic lesion according to the degree of coronary artery luminal narrowing and the location of the lesion. The total score was calculated as a sum of the product of the stenosis and location score of each affected lesion.

In this scoring system, 0 indicates no abnormality, 1 represents stenosis of ≤ 25%, 2 represents stenosis of 26–50%, 4 represents stenosis of 51–75%, 16 represents stenosis of 76%–99%, and 32 represents complete occlusion. The score is then multiplied by different factors according to the functional significance of the coronary artery. The evaluation of each segment was performed by multiplying the scores by 5 for the left main trunk, by 2.5 for the proximal left anterior descending (LAD) branch, by 1.5 for the middle LAD, by 1 for the distal LAD, by 1 for the first diagonal branch, by 0.5 for the second diagonal branch, by 2.5 for the proximal LCx, by 1 for the distal LCx and posterior descending branch, and by 0.5 for the posterior branch, while the RCA was performed by multiplying the scores by 1 for the proximal, middle and distal RCA and the posterior descending branch, and by 0.5 for the posterior branch. The final score was calculated by adding the scores of each segment. The patients were then divided according to the median of the total score (GS = 40) [12].

#### Serum interleukin-6 measurement

Serum interleukin-6 (IL-6) levels were measured by electrochemiluminescence immunoassay (ECLIA) using a Cobas E601 analyzer (Roche Diagnostics, Germany).

#### Laboratory evaluation

Serum samples were collected from study participants immediately upon admission, and stored in aliquots at -80 °C until use. Triglycerides (TG), total cholesterol (TC), high density lipoprotein-cholesterol (HDL-C), hsCRP, glucose, urae, creatinine, creatine phosphokinase (CPK) and creatine kinase (CK)-MB isoforms were measured using an analyser (Cobas Integra 600, Roche Diagnostic, Germany) for patients upon admission. Low density lipoprotein-cholesterol (LDL-C) was estimated by the Friedewald equation) [10].

#### Statistical analysis

Data were analyzed using Statistical Package for the Social Sciences (SPSS, version 23.0). Continuous variables were described as mean ± standard deviation (SD) for normally distributed data or medians (minimum—maximum) for non-normally distributed data, as appropriate. Categorical data were summarized as frequencies or percentages. IL-6 was log transformed before analyses because its non-normally distribution. Differences in quantitative parameters between groups were performed using independent-samples T test or Mann–Whitney U test, as appropriate. The predictive values of different biomarkers for the presence of CAD and evaluating infarct size were determined by constructing receiver operating characteristic (ROC) curves and the area under the curve (AUC) was calculated. *p* value < 0.05 were considered as significant. The cut-off value was determined using the Youden index = maximum (sensitivity + specificity – 1) [12].

## Results

### Baseline clinical and laboratory characteristics

The baseline clinical and laboratory characteristics of the subjects were shown in Table 1. Patients had 60.3 ± 11.0 years as mean age and were mostly males. As prevalences were high, many risk factors contributed to CAD outcomes development.

**Table 1:** Baseline characteristics of the study population

Characteristics	Controls *N* = 207	Patients *N* = 310	*p*
Age, years	33.2 ± 12.2	60.3 ± 11.0	< 0.001
Male, n (%)	61(29.5)	232(74.8)	< 0.001
BMI, Kg/m^2^	25.1 ± 3.6	27.5 ± 4.3	< 0.001
Smoker, %	8.2	41.0	< 0.001
Menopause, %	0	90.8	< 0.001
Obesity, %	0	22.6	< 0.001
Hypertension, %	0	47.1	< 0.001
Diabetes, %	0	48.4	< 0.001
Dyslipidemia, %	0	23.5	< 0.001
Personal history of CAD, %	0	33.2	< 0.001
Personal history of ACS, %	0	27.4	< 0.001
Peripheral artery Diseases, %	0	1.6	< 0.001
History of bypass surgery, %	0	3.2	< 0.001

Data are expressed as mean ± SD or n (%)

*ACS* Acute coronary syndrome, *BMI* Body mass index, *CAD* Coronary artery diseases

#### Statistical differences between biochemical markers and IL-6 levels

The cutoff value of IL-6 was determined using ROC curve analysis between all patients and controls. Based on IL-6 cutoff value, patients were separately divided into two groups, low- and high IL-6 levels. The biochemical analyses of taken blood samples were compared between both group low- and high IL-6 serum levels. Lipid profile parameters and atherogenic index levels were more elevated in patients with high levels of IL-6. Differences were not statistically significant (Table 2).

**Table 2:** Clinical, angiographic and biochemical characteristics of patients

Characteristics	All *N* = 310	Patients		*p*
		Low < 9.5 pg/mL *N* = 164	High ≥ 9.5 pg/mL *N* = 146	
SBP, cmHg	13.0 ± 2.2	12.8 ± 1.9	12.7 ± 2.3	0.818
DBP, cmHg	7.4 ± 1.3	7.4 ± 1.1	7.4 ± 1.5	0.750
Heart rate, b/min	75.5 ± 15.7	73.9 ± 14.4	77.1 ± 17.7	0.208
Ejection Fraction, %	49.3 ± 13.7	52.9 ± 13.0	45.8 ± 13.8	0.077
Vessel involved, %				
LMCA	6.9	45.5	54.5	0.581
LCx	48.4	53.2	46.8	0.959
IVA	73.0	53.4	46.6	0.996
RCA	47.2	56.0	44.0	0.544
Stenosis, %				0.723
[50–70]	26.9	57.1	42.9	
> 70	73.1	52.6	47.4	
Gensini Score categories				0.022
Low (GS < 40)	42.9	50.0	50.0	
High (GS > 40)	57.1	34.3	65.7	
Vessel Disease, %				0.344
One Vessel	44.7	49.3	56.8	
Multi Vessel	55.3	50.7	43.2	
Glucose, mmol/L	7.9 (3.1–42.3)	8.09 (3.8–21.23)	7.6 (3.1–41.6)	0.680
HbA1c, %	7.9 ± 2.4	7.9 ± 2.3	7.2(5.3–12.7)	0.630
Urae, mmol/L	5.7 (2.5–30.5)	5.2 (2.5–12.6)	5.6 (2.3–30.5)	0.003
Creatinin, µmol/L	88.0(5–164)	85(5–148)	89.5(40–164)	0.153
Uric Acid, µmol/L	309.0 (82.0–817.0)	312.0 (217.0–633.0)	316.0 (126.0–591.0)	0.051
TC, mmol/L	4.4 ± 1.4	4.4 ± 1.4	4.3 ± 1.4	0.542
TG, mmol/L	1.4 (0.3–12.1)	1.4 (0.3–12.1)	1.4 (0.5–4.8)	0.389
HDL-C, mmol/L	0.9 (0.23–1.9)	0.9 (0.23–1.83)	0.8 (0.33–1.9)	0.802
LDL-C, mmol/L	2.7 (0.4–6.0)	2.4 (0.4–5.0)	3.0 (0.6–5.0)	0.056
Lp(a), mg/dL	11.0 (1.5–123.0)	10.7 (2.3–123.0)	10.7 (1.5–110.7)	0.925
ApoA1, mg/dL	95.9 (0.0–145.0)	94.05 (0.0–145.0)	94.05 (20.1–147.0)	0.466
ApoB, mg/dL	72.2(7.2–152.0)	70.5(22.0–133.0)	74.5(7.2–152.0)	0.120
AIP	0.18(0.58–1.41)	0.15(0.35–1.41)	0.19(0.58–0.66)	0.545
ApoB/ApoA1	0.8(0.1–5.1)	0.7(0.2–3.1)	0.8(0.1–5.1)	0.075
HDLc/ApoA1	0.9(0.3–2.4)	1.0(0.3–2.4)	0.9(0.5–1.8)	0.378
HDLc/Lp(a)	7.2(0.7–58.8)	7.4(0.7–0.9)	6.5(0.9–41.7)	0.232
Hcy, µmol/L	18.9(3.7–50.0)	15.4(7.7–50.0)	23.5(3.7–50.0)	0.125
HGB, g/dL	13.2(8.1–17.4)	13.8(8.8–17.3)	13.0(8.1–17.4)	0.019
IL-6, pg/mL	9.0(1.5–3640.0)	4.6(1.5–9.4)	24.8(9.6–3640.0)	0.001
hsCRP, mg/L	4.9(0.2–1020.0)	3.2(0.2–100.0)	14.3(0.3–1020.0)	0.002
GS	53.0 ± 38.7	46.7 ± 37.2	60.6 ± 39.5	0.027

*AIP* Atherogenic index of plasma (TG/HDLc), *ApoA-1* Apolipoprotein A-1, *ApoB* Apolipoprotein B, *GS* Gensini score, *HbA1c* Gyrated hemoglobin, *Hcy* Homocysteine, *HDL-c* High density lipoprotein cholesterol, *HGB* Hemoglobin, *Hs-CRP* High sensitivity C-reactive protein, *IL-6* Interleukin 6, *LDL-c* Low density lipoprotein cholesterol, *Lp(a)* Lipoprotein(a), *TC* Total cholesterol, *TG* Triglycerides. Values are expressed as the mean ± SD. *ACE* Angiotensin converting enzyme inhibitor, *DBP* Diastolic blood pressure, *LAD* Left anterior descending, *LCx* Left circumflex, *LDL-c* Low density lipoprotein cholesterol, *LMCA* Left main coronary artery, *LVEF* Left ventricular ejection fraction, *RCA* Right coronary artery, *SBP* Systolic blood pressure. Values are expressed as the mean ± SD or n (%). *p* < 0.05. *p* < 0.05

#### Severity of coronary artery disease

GS levels were significantly more elevated in patients with high levels of IL-6 than in patients with low levels of IL-6 (60.6 ± 39.5 *vs.* 46.7 ± 37.2; *p* = 0.027) (Table 2). 64.9% of patients using statins, had IL-6 levels < 9.5 pg/mL. In high levels of IL-6 (≥ 9.5 pg/mL) patient group, 65.7% of patients had GS value > 40 (*p* = 0.022) (Table 2). High levels of IL-6 were significantly associated with high levels of GS (> 40) (*p* = 0.027) and not with stenosis degree or vessel score (Table 3).

**Table 3:** IL-6 levels distribution according to angiographic profile

Characteristics	IL-6, pg/mL		*p*
	Median	Min—Max	
Stenosis			
[50–70] %	9.1	1.8–203.3	0.783
> 70%	7.7	1.5–3640.0	
Number of stenotic coronary arteries			
Single vessel diseased	9.6	1.8–1957.0	0.274
Multi-vessel diseased	8.1	1.5–3640.0	
Gensini Score categories			
Low (GS < 40)	7.1	2.1–1957.0	0.027
High (GS > 40)	10.9	2.2–3640.0	

Values are expressed as the mean ± SD.* p* < 0.05

*Abbreviations*: *GS* Gensini score, *IL-6* Interleukin 6, *Max* Maximum value, *Min* Minimum value

**Fig. 1:**
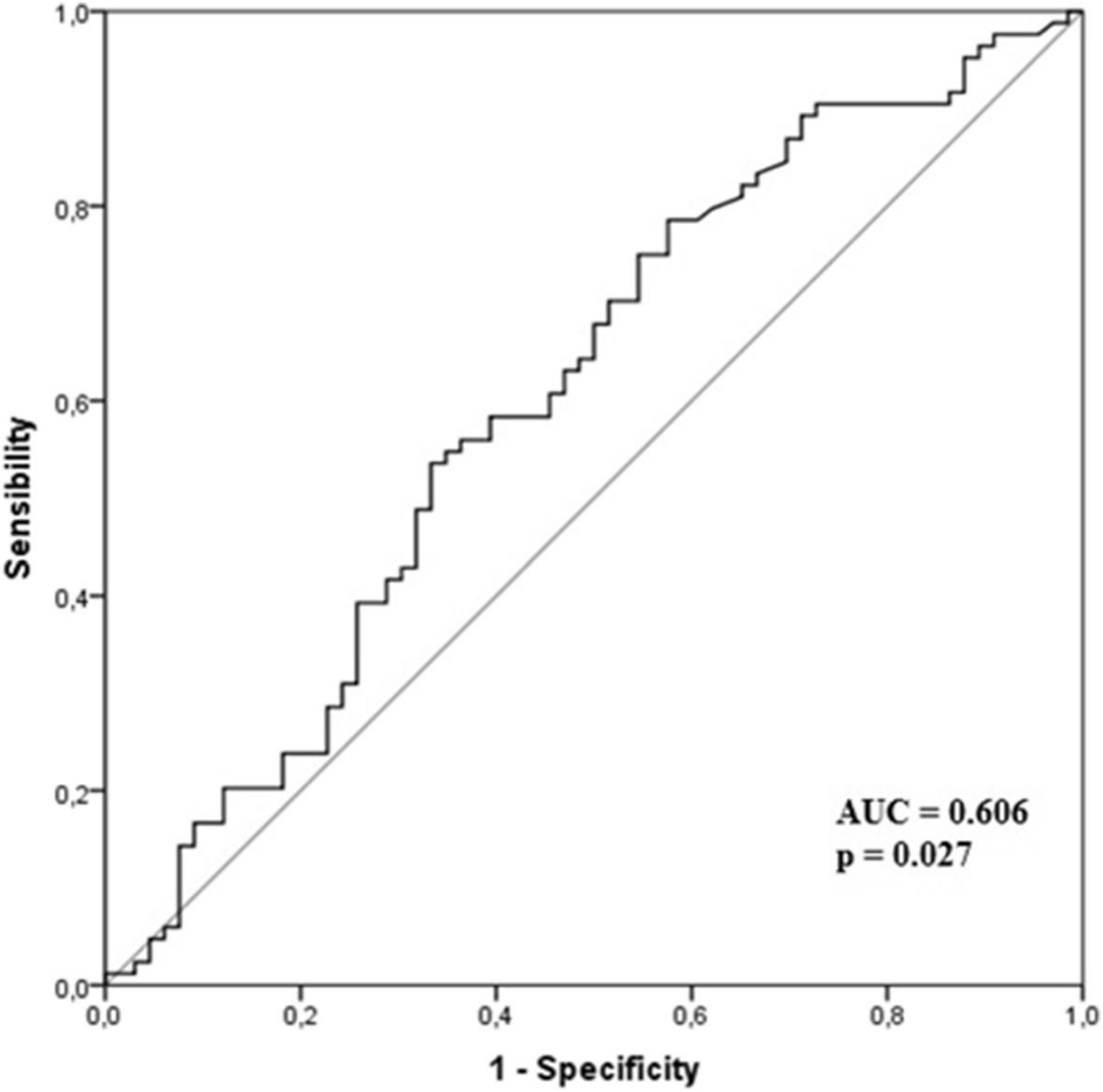
ROC analysis for IL-6 levels in predicting CAD severity performed in patients with GS > 40. Area under the ROC curve for IL-6 levels was 0.606; CI: 0.513 – 0.698, *p* = 0.027

IL-6 was a predictor of CAD severity according to the ROC Analysis performed in patients with GS > 40 [area under the curve (AUC) 0.606; CI: 0.513 – 0.698, *p* = 0.027] (Fig. 1).

#### IL-6 levels in different ACS patients subgroups

The subgroup analysis of all patients divided into the NSTE-ACS group, STEMI group and UA showed that serum IL-6 levels in STEMI patients were significantly higher than levels in other patients (19.1 pg/mL vs. 7.5 pg/mL (NSTEMI) and 5.6 pg/mL (UA) (Table 4).

**Table 4:** Levels of IL-6 in different clinical groups

IL-6, pg/mL	Controls *N* = 207	All patients *N* = 310	*p*	UA *N* = 107	NSTEMI *N* = 111	STEMI *N* = 92
Median V Min; V Max	3.1 1.1–8.7	8.9 1.5–3640.0	< 0.001	5.6 1.7–855.4	7.5 1.5–220.0	19.1 2.4–3640.0

*Abbreviations*: *IL-6* Interleukin 6, *NSTEMI* Non ST elevation myocardial infarction, *STEMI* ST elevation myocardial infarction, *UA* Unstable Angina, *V max* Maximum value, *V min* Minimum value, *p* (NSTEMI *vs* UA) = 0.108; *p* (STEMI *vs* UA) < 0.001; *p* (NSTEMI *vs* STEMI) < 0.001. *p* values < 0.05 statistical signifiance

#### Prediction of infarct size in patients with MI by ROC curve analysis

**Fig. 2:**
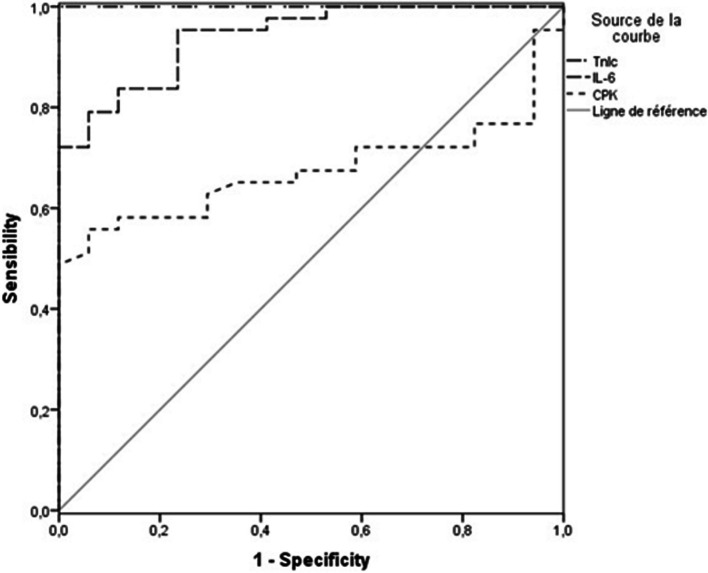
The analysis by ROC curve for IL-6, TnIc and CPK. AUC for IL-6 = 0.941 (CI 95% 0.886, 0.997; *p* < 0.001); CPK: 0.674 (CI 95% 0.543–0.806; *p* = 0.036); cTnI 1.00 (CI 95% 1.000, 1.000; *p* < 0.001)

The analysis by ROC curve for IL-6, TnIc and CPK showed an AUC for IL-6 = 0.941 (CI 95% 0.886, 0.997; *p* < 0.001); CPK: 0.674 (CI 95% 0.543—0.806; *p* = 0.036); cTnI 1.00 (CI 95% 1.000, 1.000; *p* < 0.001) (Fig. 2). These results suggest that IL-6 could be a powerful marker in predicting infarct size in STEMI patients.

## Discussion

In this study, we investigated the relationship between serum IL-6 levels and the severity of coronary artery disease (CAD). Our results demonstrated that IL-6 levels were associated with Gensini score in CAD patients. We found that IL-6 levels were significantly elevated in the group of patients with high GS in comparison with low GS group. IL-6 levels, as shown in ROC curve, could be a useful tool in the prediction of CAD.

Our findings were in accordance with other previous studies. In this study, the main results were the significant association between IL-6 and the severity of CAD assessed by GS. A serum IL-6 level above 9.5 pg/mL is highly predictive for severe CAD. Our findings could suggest a different treatment approach between patients on the basis of the CAD severity assessed by GS. Tanindi et al. [13] had found that IL-6 was associated with more extensive and severe CAD as represented by GS. Despite significant association between high levels of IL-6 in single- and multi vessel disease groups as compared with controls, Mokhtar ER et al. [14] findings did not show significant association between IL-6 and GS. Gotsman I et al. [15] reported that there was significant correlations between interleukin-6 with the severity of CAD assessed by the number of obstructed coronary vessels and the lesions severity. Also, Chun-lin L et al. [16] reported that IL-6 could be a useful marker in evaluating the activity of plaque and CAD degree.

Atherosclerosis depends on several well-established risk factors. Several inflammatory markers could stratify patients with high CV risk. Inflammation and the altered release of cytokines are the processes promoting the formation, development and rupture of atherosclerotic plaque which is the essential mechanism causing ACS [17]. IL6 is synthesized by several cell types such as hepatic fibroblasts, macrophages, and monocytes as well as fat cells, vasculature and myocardial cells [18]. A previous study suggests that the important secretion of IL-6 by adipose cells could explain CAD occurrence [19]. It has been shown that in large populations, IL6 compared to other inflammatory markers such as hsCRP and LpPLA2, appears to be the most predictive marker of clinical manifestations even at early stages. Karabağ Y. et al. [20] found that C-Reactive Protein/Albumin Ratio was more tightly associated with the complexity and severity of CAD than CRP and albumin alone and was found to be an independent predictor for intermediate-high Syntax Score patients. High levels of IL-6 suggest a predictor role of major cardiovascular events in CAD patients and healthy subjects [21]. IL-6 levels were associated with cardiovascular complications of MI [22], which support its role as potent activators of local inflammation [23] and could explain high levels of IL6 shown in STEMI patients.

High concentrations of IL-6 have been associated with acute ischemia and may be markers of recurrent CAD [24]. Other studies have shown that IL-6 and its different signaling pathways involve different mechanisms to contribute to the formation and destabilization of atherosclerotic plaques [25]. Once released into the blood, IL-6 molecules activate inflammatory cells, release chemokine and adhesion molecules, and triggers aggregation of these cells to form atherosclerotic plaque. The release of oxygen free radicals and the excessive expression of metalloproteinases by activated inflammatory cells lead to the instability of the atherosclerotic plaque. Secondly, the complement system could be activated also by IL-6 and affect the synthesis of endothelin-1 (ET-1) and nitric oxide altering subsequently the endothelial function. Finally, IL-6 might promote the expression of the tissue factor type I plasminogen activator inhibition factor and intensify its activities, inducing coagulation abnormalities and thrombus formation [26]. High blood levels of IL-6 may have an important role in the transformation of macrophage to foam cells in atherosclerosis in CAD patients.

In the vascular tissue, hypercholesterolemia was associated with the presence of an inflammatory reaction. Oxidized LDL, which is the inflammatory agent [27], had a chemo-attracting power on monocytes, cause their differentiation into macrophages and inhibit their migration [28]. In patients with CAD, statins have been shown to possess anti-inflammatory properties as the decrease of inflammatory cells number by inhibiting adhesion molecules and cytokines as interleukins 6 and 8 [29, 30]. These processes explain high levels of lipids and their ratios in patients with high levels of IL-6. A previous study reported that in AMI patients, IL-6 levels began to increase 14 h (mean; range 8–20 h) after the initial complaints and reached maximal levels of 28 to 250 U/ml (normal values < 10 U/ml) after 36 h (mean; range 24–52 h). This study suggest that a decrease of IL-6 levels 48 h after admission was associated with an uneventful course, and an elevation at this time point was associated with a complicated hospital course [31]. Ikonomidis et al. [32], findings suggested that therapeutic effects of aspirin and statins could explain the decrease of IL-6 level in patients with ACS. These findings could explain the large interval of IL-6 distribution in our patients.

In STEMI patients, circulating levels of IL-6 have been shown to correlate with high levels of cTnI thereafter, with myocardial necrosis size [7]. As shown in ROC curve analysis in this study, IL-6 and necrosis markers were grateful tools in predicting infarct size. Pre-inflammatory cytokines could be involved in reperfusion injury, repair, and healing processes following MI [7].

However, our study also has some limitations. First, coronary angiography detects only coronary luminal narrowing and not systemic atherosclerotic burden; the latter may affect serum levels of inflammatory markers. Second, IL-6 levels were measured in peripheral blood; this does not elucidate the site of origin of inflammatory mediators. Third, despite the common pathophysiology process, CAD outcomes differ in clinical symptoms, which distort results.

Fourth, we did not collect the time of onset of chest pain the samples for IL-6 were taken which may influence the IL-6 levels. Future studies will need to assess the importance of other parameters (i.e., genetic factors, novel risk factors) in angiographic CAD extent prediction. These results apply only to the angiographic assessment of the presence, severity, and extent of CAD; other imaging techniques such as intravascular ultrasound or optical coherence tomography may yield different results.

## Conclusions

Our results suggest that IL-6 is useful to be a good biomarker reflecting the severity of CAD assessed by GS and to play a major role in the progression of atherosclerosis. Based on the highest levels of IL-6 measured in patients with STEMI, our study strongly suggests that IL-6 could be a powerful marker in evaluating the necrosis extent and high levels could be related to post MI complication.

## Data Availability

The data used to support the findings of this study are available from the corresponding author upon request.

## Abbreviations

ACS: Acute coronary syndrome
CAD: Coronary artery disease
CEI: Conversion enzyme inhibitor
CK-MB: Creatine kinase isoforms
CPK: Creatine phosphokinase
cTnI: Cardiac troponin I
DBP: Diastolic blood pressure
ECLIA: Electrochemiluminescence immunoassay
ESC: European Society of Cardiology
ET: Endothelin-1
GS: Gensini score
HDL-c: High density lipoprotein-cholesterol
HsCRP: High sensitivity C reactive protein
IL: Interleukin
LAD: Left anterior descending
LCx: Left circumflex
LDL-c: Low density lipoprotein-cholesterol
LMCA: Left main coronary artery
LpPLA2: Lipoprotein-associated phospholipase
LVEF: Left ventricular ejection fraction
MI: Myocardial infarction
NSTEMI: Non-elevation myocardial infarction
RCA: Right coronary artery
SBP: Systolic blood pressure
STEMI: ST-segment elevation myocardial infarction
TC: Total cholesterol
TG: Triglycerides
UA: Unstable angor
